# Metformin and breast cancer: an opportunity for pharmacogenetics

**DOI:** 10.18632/aging.204180

**Published:** 2022-07-18

**Authors:** Elisabet Cuyàs, Begoña Martin-Castillo, Javier A. Menendez

**Affiliations:** 1 Metabolism and Cancer Group, Program Against Cancer Therapeutic Resistance, Catalan Institute of Oncology–Girona Biomedical Research Institute, Girona, Spain; 2 Clinical Research Unit, Catalan Institute of Oncology–Girona, Girona, Spain

**Keywords:** metformin, breast cancer, single-nucleotide variant, type 2 diabetes, HER2

A potential beneficial effect of the anti-diabetic biguanide metformin across several cancer types has gained observational and pre-clinical support over the last decade. The initial enthusiasm arising from the cancer–preventive outcomes associated with metformin usage among patients with type 2 diabetes (T2D) has been gradually tempered after disappointing results in randomized clinical trials testing the therapeutic efficacy in non-diabetic patients with established cancers, particularly those with advanced disease. Most researchers in the field have been eagerly expecting the results from the NCIC CTG MA.32 study, the largest clinical trial investigating whether the use of metformin (vs. placebo) given for 5 years would improve invasive disease-free survival (IDFS) and other outcomes in non-diabetic women with high-risk operable breast cancer. The long-awaited results from the MA.32 study, an investigator-initiated phase 3, placebo-controlled, double-blind multinational trial enrolling 3,649 breast cancer (BC) patients that were randomized to receive 850 mg of oral metformin twice a day (*n* = 1,824) or oral placebo twice a day (*n* = 1,825) for 5 years beginning after completion of definitive therapy, have concluded that the addition of metformin vs. placebo to standard BC treatment did not significantly improve invasive disease-free survival (IDFS) among patients with high-risk operable BC without diabetes [1].

The findings of the MA.32 trial do not support adding metformin to standard adjuvant therapy in non-diabetic BC patients with early-stage disease. However, they should not be considered negative [2]. First, the results offered by the MA.32 study do not preclude the use of metformin to treat T2D in BC patients, which might alleviate some of the multi-faceted effects of diabetes in BC prognosis. Second, the findings of the trial appear to support the idea that metabolically healthy individuals could not benefit from the potential anti-cancer effects of metformin. Third, and most importantly, we now dispose of strong hypothesis-generating data about the beneficial impact of metformin in human epidermal growth factor receptor 2+ (HER2+) BC patients, particularly among those with any *C* allele of the rs11212617 single-nucleotide variant (SNV, formerly single-nucleotide polymorphism [SNP]). The exploratory analysis of the MA.32 trial in the HER2+ population was informed by the METTEN study [3], a phase 2 trial of neoadjuvant metformin in combination with trastuzumab and chemotherapy in early HER2+ BC conducted by us in 2018. Although underpowered, the METTEN study demonstrated higher pathological complete response (pCR) rates and more frequent use of breast conservation surgery among those HER2+ patients allocated metformin [3]. In a follow-up substudy, we reported that adding metformin led to significantly >2–fold higher rates of pCR in HER2+ BC patients with at least 1 copy of the minor rs11212617 *C* allele but not among those with the *AA* genotype [4]. In the metformin-containing arm, the positive association between the presence of the rs11212617 *C* allele and the probability of achieving a pCR remained significant after accounting for potential confounding tumor characteristic such as tumor size and hormone receptor status. The exploratory analyses in the HER2+ subpopulation in the MA.32 trial –a majority of whom received trastuzumab adjuvant therapy– demonstrates that metformin associates with longer IDFS and overall survival (OS) in those patients with any *C* allele of the rs11212617 SNV (*P* for interaction = 0.05 and 0.02, respectively) [1].

**Figure 1:**
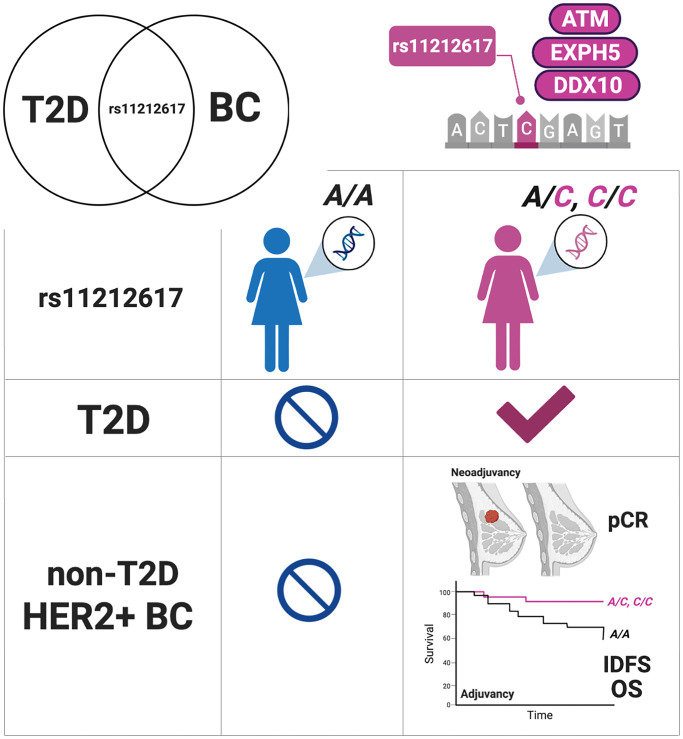
**The *C* allele of the rs11212617 SNV: a pharmacogenetic biomarker of response to metformin at the interface of T2D and HER2+ breast cancer.** rs1121261 should be explored in prospective trials as a pharmacogenomic profiling system to individualized metformin therapy for breast cancer patients. (Abbreviations: T2D: type 2 diabetes; BC: breast cancer; pCR: pathological complete response; IDFS: invasive disease-free survival; OS: overall survival). Created with https://Biorender.com.

Only non-diabetic (type 1 or 2) HER2+ breast cancer patients without metabolic comorbidities at baseline (e.g., obesity, impaired glucose tolerance) were eligible for the METTEN study [3]. Therefore, it must be emphasized that a “favorable” *C* allele-containing rs11212617 genotype –an allele previously associated with treatment success on metformin in T2D patients (Figure 1) – is a predictor for achieving pCR in non-diabetic HER2+ BC patients. In our hands, a nonsignificant trend toward significance of reduced circulating insulin and HOMA-IR index (*p* = 0.069 and 0.093, respectively) occurred in those HER2+ BC patients with any *C* allele of the rs11212617 SNV treated with metformin. The rs11212617 SNV is located in a large linkage disequilibrium block involving an *ATM* intronic enhancer and several target genes such as *EXPH5* and *DDX10* that have been associated with insulin resistance [5]. It will be essential to disentangle which candidate(s) might alter metabolic health in BC patients that may benefit most from metformin (e.g., at risk of developing T2D or with alterations in other metabolic parameters). Given the absence of a significant interaction of HER2 status with metformin effect in the MA.32 trial [1], if the presence of the *C* allele of the rs11212617 SNV might associate with a potential metformin benefit in HER2-negative BC should be explored. Nonetheless, confirmation that the association between the rs11212617 *C* allele and the benefit of adding metformin in terms of clinical outcomes (pCR, IDFS, OS) is equivalent in neoadjuvant and adjuvant treatments of non-diabetic HER2+ BC patients should be viewed as strong-hypothesis-generating data that warrants replication in a prospective trial to replicate if the pharmacogenomic profile of the rs11212617 SNV genotype should definitely deserve consideration as a predictive biomarker to inform the use of metformin in non-diabetic BC patients.
